# Molecular phylogeny of a novel human adenovirus type 8 strain causing a prolonged, multi-state keratoconjunctivitis epidemic in Germany

**DOI:** 10.1038/srep40680

**Published:** 2017-01-13

**Authors:** Elias Hage, Werner Espelage, Tim Eckmanns, Daryl M. Lamson, Laura Pantó, Tina Ganzenmueller, Albert Heim

**Affiliations:** 1 Institute of Virology, Konsiliarlabor für Adenoviren (KLA, Adenovirus Reference Laboratory); Hannover Medical School, Hannover, Germany; 2 Department for Infectious Disease Epidemiology, Unit for Nosocomial Infections, Surveillance of Antimicrobial Resistance and Consumption, Robert Koch Institut, Berlin, Germany; 3 Wadsworth Center, New York State Department of Health, Albany, NY, USA; 4 Laboratory of Genome Sciences, Division of Bioengineering and Bioinformatics, Graduate School of Information Science and Technology, Hokkaido University, Sapporo, Japan

## Abstract

The German infectious disease surveillance system revealed an increase of epidemic keratoconjunctivitis (EKC) from an average of 320 cases/year (2001 to 2010) up to 2146 and 1986 cases in 2012 and 2013, respectively. From November 2011 until December 2013 (epidemic period) 85% of typed isolates were human adenovirus type 8 (HAdV-D8), whereas only low level circulation (19%) of HAdV-D8 was observed outside the epidemic period. In order to investigate whether a novel monophyletic HAdV-D8 strain prevailed during the epidemic period, complete genomic sequences of 23 HAdV-D8 isolates were generated by deep sequencing and analyzed phylogenetically. For comparison, eight HAdV-D8 isolates from outside the epidemic period were sequenced. HAdV-D8 isolates of the epidemic period had a very high sequence identity of at least 99.9% and formed a monophyletic cluster with two subclusters. A single outlier was closely related to HAdV-D8 strains isolated prior to the epidemic period. Circulation of the epidemic strain was detected as early as 2010 but not after the epidemic period in 2014. In conclusion, molecular phylogeny of complete genomic sequences proved a monophyletic HAdV-D8 epidemic. However, co-circulation of other HAdV types as well as better reporting may have contributed to the huge increase of reported cases.

Human adenoviruses (HAdV), which comprise 70 types classified into seven species (A–G), are common pathogens causing respiratory, gastrointestinal, genitourinary and ocular diseases^1^. Epidemic keratoconjunctivitis (EKC) is a severe inflammatory ocular disease affecting both the conjunctiva and the cornea^2^. Inflammation of the cornea can lead to impaired vision for months to years following the infection. EKC is caused by only five of the 45 types of the species HAdV-D: HAdV-D8, HAdV-D64 (previously known as genome type HAdV-D19a), HAdV-D37, HAdV-D53 and HAdV-D54^3^,^4^,^5^,^6^. EKC-associated type HAdV-D37 (and probably all EKC associated types) use α2,3-linked sialic acid as primary receptor^7^,^8^ instead of the coxsackie-adenovirus receptor (CAR), which is used by most other HAdV types^9^. Occasionally other types (e.g. HAdV-E4, HAdV-B3, HAdV-D56) can be isolated from keratoconjunctivitis and conjunctivitis cases^2^.

Because of the severity and public health impact of EKC, notification to local health authorities is mandatory for laboratories that detect adenoviruses from conjunctival swabs according to the German Protection against Infections Act (IfSG, §7.1)^10^ and the Robert Koch Institute (RKI) case definitions^11^. Notifications are transmitted via local health authorities and the federal state authorities to RKI according to the IfSG. Besides, reporting of nosocomial outbreaks with the same reporting path is mandatory (IfSG §6.3). The German notification system has been described previously in detail^12^.

Starting with a big nosocomial EKC outbreak caused by HAdV-D8 in November 2011^13^, adenovirus (kerato-) conjunctivitis case numbers reported to RKI increased from an average of 320 cases/year in the years 2001 until 2010 to 2146 cases in 2012 (Table 1). Circulation of HAdV-D8 had not been observed in Germany from 2006 to 2009. Epidemiological data collected according to the IfSG were analyzed together with results of the reference laboratory (KLA, Konsiliarlabor für Adenoviren at the Hanover Medical School). Complete adenovirus genomic sequences of 31 EKC cases were generated in order to investigate that the circulation of a monophyletic HAdV-D8 strain caused the prolonged multi-state EKC epidemic observed in 2012 and 2013 (analyzed epidemic period from November 2011 to December 2013).

## Results

### Cases reported to Robert Koch Institute 2001–2015

In total 3723 outbreak related cases and 6009 sporadic cases were reported to RKI in the period from January 2001 until December 2015. Of notice is a sharp increase in the number of both sporadic cases and outbreak cases in the year 2012 and 2013 with a maximum of 2146 reported cases in 2012 and 1986 in 2013, corresponding to an incidence of 2.6 and 2.4/100,000 inhabitants, respectively (Table 1). Among 300 typed isolates from 2001 to 2015, 119 (39.7%) were identified as HAdV-D8 (Table 2). Other reported types were HAdV-D64 in 139 cases (46.3%), HAdV-B3 in 27 cases (9.0%), HAdV-D37 in 8 cases (2.7%), HAdV-B7 in 2 cases (0.7%) and HAdV-E4 in 5 cases (1.7%). During the epidemic period however, HAdV-D8 cases significantly predominated compared to the non-epidemic period (Table 2, 79 of 93 typed cases, vs. 40 of 207 typed cases; p < 0.0001 Fisher’s exact test).

From November 2011 until December 2013 (epidemic period), fourteen HAdV-D8 outbreaks were notified to the RKI. Cases epidemiologically linked (“epi”) to a laboratory confirmed (“lab”) case were considered to be of the same type. Federal States affected by these HAdV-D8 outbreaks were Baden-Wurttemberg (4 outbreaks with 2, 2, 9 and 15 cases, respectively; 16 of these lab, 12 epi), Bavaria (4 outbreaks with 2, 3, 6 and 27 cases, respectively; 7 of these lab, 31 epi), Lower Saxony (2 outbreaks with 3 and 47 cases, respectively; 4 of these lab, 46 epi), Mecklenburg-Vorpommern (1 outbreak with 3 cases, all lab) and North Rhine-Westphalia (3 outbreaks with 6, 24 and 209 cases, respectively; 10 of these lab, 229 epi). Another 39 sporadic HAdV-D8-cases (i.e. cases without other epidemiologically linked cases) were reported in the epidemic period. For comparison, only three HAdV-D8 outbreaks with 58 cases were observed outside the epidemic period (2001 to October 2011, 2014 and 2015) as well as 8 sporadic cases.

Patients infected with HAdV-D8 were significantly older and more often female than patients infected with other types (Table 3). Information on the presumed place of infection (setting) was provided only for 236 HAdV-D8 outbreak-cases and for 1187 other outbreak cases. Infections were considered healthcare-associated when the assumed place of infection was practice, medical treatment center or hospital. In contrast to outbreak cases caused by other or unknown types, most HAdV-D8 outbreak cases (233 of 236 (98.7%) with information, including all 209 cases in the large outbreak in North Rhine-Westphalia, November 2011) were healthcare associated (Table 3).

Of all HAdV-D8 outbreak cases reported to RKI from 2001 until 2015, 86.1% were reported during the epidemic period.

### Epidemic HAdV-D8 cases analyzed at the reference lab (KLA)

HAdV DNA was detected in 167 cases sent to the KLA in the epidemic period. Molecular typing was achieved in 120 cases directly by sequencing from the diagnostic specimen but failed in 47 cases due to technical reasons (e.g. insufficient HAdV DNA concentration). HAdV-D8 was identified in 99 specimens (82.5%), HAdV-D37 in 9 specimens (7.5%), HAdV-D53 and HAdV-E4 in 4 specimens (3.3%) respectively, and HAdV-B3 and HAdV-D19a ( = D64) in 2 specimens (1.7%), respectively.

Because reporting to the RKI is anonymous and a common identifier of cases was missing, it was not possible to merge the RKI data set with the KLA laboratory results. In both data sets, HAdV-D8 clearly predominated during the epidemic period: 99 of 120 (82.5%) KLA cases were typed as HAdV-D8, compared to 79 of 93 (84.9%) in the RKI data set mentioned above (Table 2). Moreover, no significant differences were found in age and gender between HAdV-D8 positive patients in the KLA data set (n = 99) and in the RKI data set (n = 79): median age 58 years (interquartile range 45–70) vs. 61 years (interquartile range 49–70) and 54.3% female cases vs. 50.6% female cases, respectively.

**Figure 1:**
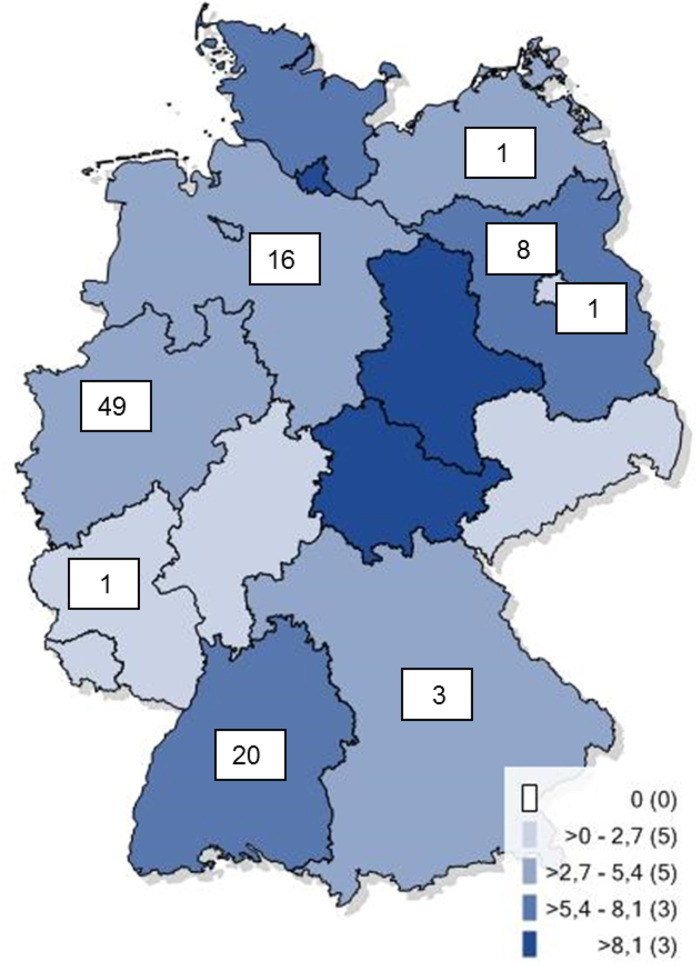
Number of HAdV-D8 cases analysed at the KLA (white boxes) and Adenovirus-(Kerato)- conjunctivitis incidence in different German states (different shades of blue, see legend) in the epidemic period. The number of federal states with the respective incidence category is given in brackets. No samples were received from the states with the highest incidences, Saxony-Anhalt and Thuringia (Base map together with incidence rates were generated and plotted using the online tool and database at Robert Koch Institute: https://survstat.rki.de/Content/Query/Main.aspx version 2.0, accessed on Jan 4, 2016).

The geographical distribution of HAdV-D8 cases available at the KLA and the incidence of EKC in the federal states (RKI data) are depicted in Fig. 1. Typed cases at the KLA originated from eight of the sixteen German states (Bavaria, Berlin, Brandenburg, Baden-Wurttemberg, Lower Saxony, Mecklenburg-Vorpommern, North Rhine-Westphalia and Rhineland-Palatinate). No samples were received at the KLA from two states with a high EKC incidence (Saxony-Anhalt and Thuringia) but also no typing results were reported from these two states to the RKI.

#### HAdV DNA quantification

Adenovirus DNA concentrations (median 3.9 × 10^7^ copies (genome equivalents)/ml, 25th percentile 5.4 × 10^6^ copies/ml, 75th percentile 2.5 × 10^8^ copies/ml) of HAdV-D8 positive samples were significantly higher (p = 0.0065, Mann-Whitney test) compared to samples containing other types (median 2.3 × 10^6^ copies/ml, 25th percentile 4.6 × 10^5^ copies/ml, 75th percentile 2.6 × 10^7^ copies/ml). All values relate to eye swab specimens resuspended in 1 ml sterile 0.9% NaCl.

#### Complete Genomic Sequences and Phylogenetic Analysis

The complete adenovirus genomic sequences of 31 HAdV-D8 cases were determined using next generation sequencing (NGS) including 23 cases from the epidemic period, 6 cases from 2003 to 2010, and two cases from 2014.

**Figure 2:**
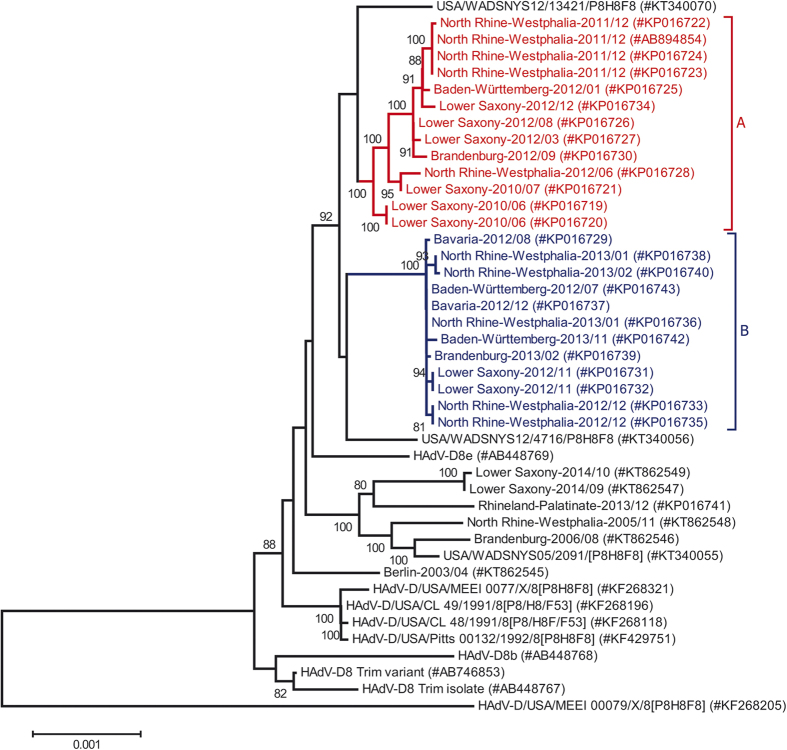
Phylogenetic analysis of the HAdV-D8 whole-genome sequences. The monophyletic cluster containing all epidemic associated sequences (except a single outlier from Rhineland-Palatinate, #KP016741) and German sequences from 2010 is highlighted, as well as subclusters A and B. The neighbour-joining tree was generated based on the Kimura two-parameter model with MEGA6. Bootstrap values < 80% are not robust and therefore not depicted.

All sequences from the epidemic period (with exception of a single outlier sequence) formed a monophyletic cluster, supported by a bootstrap value of 92% (Fig. 2). This monophyletic cluster consisted of two subclusters (A and B) and both were supported by bootstrap values of 100%. Subcluster A included sequences from the early epidemic period, with the last sequence from December 2012. In addition, subcluster A included all sequences from the November/December 2011 outbreak in North Rhine-Westphalia and the last pre-epidemic German sequences from Lower Saxony, 2010. Sequence diversity was higher in subcluster A (maximum diversity 0.07%) than in subcluster B (maximum diversity 0.02%). Circulation of subcluster B HAdV-D8 strains (first sample isolated in July 2012) overlapped with circulation of subcluster A HAdV-D8, both temporally and geographically. Two American HAdV-D8 strains isolated in 2012 (#KT340070 and #KT340056) clustered with the German sequences of the 2012/2013 epidemic but were neither associated with subcluster A nor with subcluster B. All these sequences were most closely related to the sequence of a clinical HAdV-D8 isolate from Japan (#AB448769.1), labelled as genome type 8e (Fig. 2).

A single outlier from Rhineland-Palatinate, December 2013 (#KP016741) did not cluster with the other sequences of the epidemic period but formed a second monophyletic cluster with German samples isolated in 2014 and prior to 2010. This cluster also included the sequence of an American HAdV-D8 strain isolated in New York state in 2005 (#KT340056).

#### In silico restriction enzyme analysis (REA)

**Figure 3:**
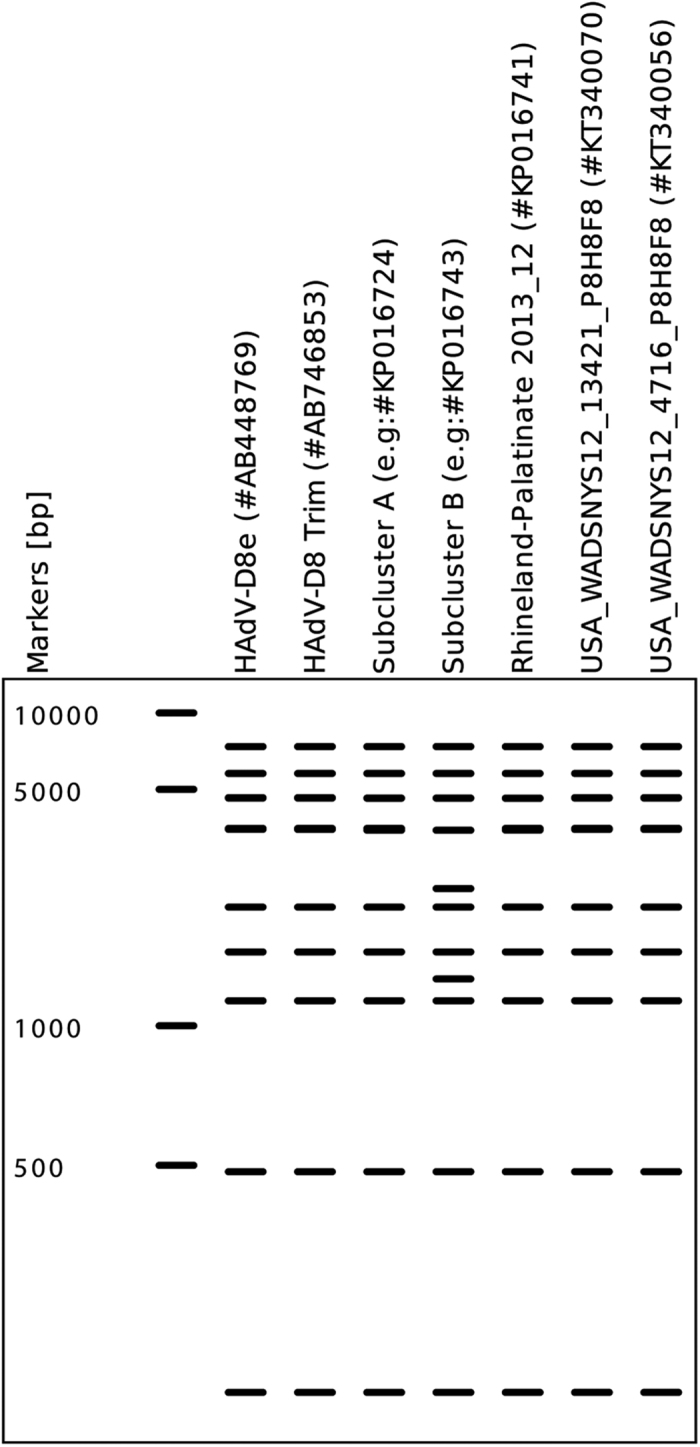
An in silico REA analysis was performed using a representative sequence from each of the subclusters (**A** and **B**) together with HAdV-D8e, Trim and the American sequences (#KT340070 and #KT340056). One additional cut site was observed in subcluster B sequences using SacI digestion. All sequences showed an identical band pattern with both HindIII and SmaI digestions (not depicted). In silico REA analysis was performed using CLC Genomics Workbench v7.

Subcluster A and the two American sequences (#KT340070 and #KT340056) that clustered with German epidemic sequences showed an identical REA pattern to genome type 8e, whereas one additional SacI cleavage site was observed in subcluster B sequences (Fig. 3). Thus, the monophyletic cluster of sequences from the epidemic period comprised two different REA patterns. On the other hand, several other sequences which formed a second monophyletic cluster with the above mentioned outlier from the epidemic period (#KP016741) had an in silico REA pattern identical to genome type 8E and the subcluster A sequences.

#### Common and unique features of the HAdV-D8 sequences from the epidemic

Genomic sequences of the monophyletic cluster from the epidemic period had 40 single nucleotide polymorphisms in common compared to the HAdV-D8 prototype (Trim strain), and revealed a very high nucleotide sequence identity (≥99.85%) to each other.

Nucleic acid sequences and deduced amino acid sequences of the major capsid proteins (penton base, hexon, fiber) were found to be highly conserved between the German epidemic period samples. Compared to the HAdV-D8 prototype, all samples had only 3 amino acid substitutions (Leu809Val, Phe811Leu, Thr819Ala) in the hexon, which were not located in the hypervariable loops of the neutralization determinant. Only a few point mutations were noted: A single sample of the November/December 2011 outbreak in Northrhine-Westphalia (# KP016722) had a non synonymous point mutation (Asp270Asn) in the loop1 of the neutralization determinant. Another sample (Brandenburg, 2012/09, #KP016730) had a single amino acid substitution (Met494Ile) in the hexon but not located in its neutralization determinant. This sample also had a non synonymous fiber mutation (Asp102His). The only other fiber mutation (Glu350Gln) was found in a sample from Lower Saxony, 2012/03 (#KP016727).

Two samples originating from Northrhine-Westphalia isolated in 2013/01 (#KP016738) and 2013/02 (#KP016740) lacked the CR1-alpha open reading frame due to a frameshift mutation caused by a 5 bp deletion, in addition to a single nucleotide insertion in the CR1-gamma (29.1 kDa protein) ORF causing a frameshift mutation and thus truncation of the protein. As these gene products are not essential for HAdV replication in cell cultures, it should be pointed out that these deletions were found in genomic sequences derived directly from eye swabs without virus isolation on cell culture.

On the other hand, the outlier sequence (#KP016741), which was not part of the monophyletic cluster of the epidemic period, had a 9 bp deletion within the coding region of the DNA binding protein (E2A genome region).

## Discussion

Although adenovirus EKC is a notifiable disease according to the German Protection against Infection Act (IfSG), underreporting is presumed as doctors often diagnose EKC solely based on clinical symptoms. However, the RKI reference definition for reporting requires additional laboratory evidence or an epidemiological link to a laboratory confirmed case.

From November 2011 to December 2013, a huge increase in notified adenovirus eye infections was observed in Germany starting with a big nosocomial outbreak in Northrhine-Westphalia affecting 209 patients^13^. This outbreak received a lot of attention in mass media. In (or after) outbreak situations, when public awareness is high, it is likely that more cases get a laboratory confirmation or will be epidemiologically linked to these cases. Preliminary investigations on the epidemic period had a confusing result, as several types of HAdV were detected (types 3, 4, 8, 64 (previously known as genome type 19a), 37, 53). This finding raised suspicion that increased laboratory testing of eye swabs picked up low level circulation of multiple HAdV types because in the previous years (2001–2010) merely one or two different HAdV types were detected per year. Therefore, increased reporting due to increased laboratory testing has to be considered when comparing case numbers of 2012 and 2013 to previous years.

However, HAdV-D8, which was only occasionally isolated in the previous years (in 2004 and 2005 but not in 2006 to 2009), predominated in about 85% of the typing results in the epidemic period from November 2011 to end of 2013. HAdV-D8 seemed to re-emerge in June 2010 but it was found to be a novel strain phylogenetically linked to the later epidemic HAdV-D8 strain. Molecular phylogeny strongly supported the epidemiological hypothesis of a prolonged, multi-state epidemic caused by a monophyletic HAdV-D8 strain between November 2011 and December 2013 in spite of co-circulation of other HAdV types. Moreover, there is a hint that HAdV-D8 strains closely related to the German epidemic HAdV-D8 strain circulated world-wide because two American sequences clustered with the German 2012/13 epidemic samples, although these American sequences were not assigned to either subcluster A or B. All these sequences were most closely related to the sequence of a Japanese isolate of the genome type 8e (#AB448769)^4^,^14^. This is the first study on molecular epidemiology of EKC analyzing complete genomic sequences.

Classical adenovirus typing approaches such as epitope sequences and restriction enzyme analysis (REA) patterns can be deduced from complete genomic sequence data sets^14^. However, REA would lead to misleading results and conclusions compared to phylogenetic analysis of complete genomic sequences. REA separated the closely related monophyletic subclusters A and B into two different genome types, and on the other hand associated the outlier sequence (#KP016741) with subcluster A. Previously, an analysis of the molecular phylogeny of HAdV-B21 also showed that very closely related isolates of a monophyletic subtype were resolved as two genome types by REA, thus erroneously obscuring a monophyletic origin^18^. Epitope sequences of the hexon (neutralization epitope) and the fiber (hemagglutination inhibition epitope) of this study were highly conserved and thus also not helpful for molecular epidemiology, as already reported by a previous review^14^.

A few German epidemic HAdV-D8 strains had additional deletions in genes coding for proteins (CR1α, CR1γ) not essential for virus replication. These may either impede the immune response (CR1α gene)^19^ or may be associated with an EKC phenotype (CR1γ gene)^20^. However, the significance of these mutations for virulence and disease severity of the epidemic HAdV-D8 strain cannot yet be estimated, mostly for the reason that adequate data for other HAdV-D8 strains were not yet available. Viral loads in eye swabs of the 2012/2013 epidemic HAdV-D8 samples were significantly higher compared to virus loads in eye infections with other HAdV types, indicating higher levels of cytolytic virus replication and infectious virus progeny. This suggested a high virulence of the epidemic HAdV-D8 strain. However, this result should be considered cautiously because the adequate control group, virus loads from non-epidemic HAdV-D8 eye swabs, was not available.

Low population immunity may have promoted the re-emergence of HAdV-D8, leading to a prolonged multi-state epidemic, but data on the immune status against HAdV-D8 were not available for the German population. Low prevalence of HAdV-D8 in the years prior to the epidemic may have resulted in low population immunity. However, this has to be interpreted with caution, as HAdV-D8 infections of children and adolescents, the presumed non-immune age groups, were not reported. Moreover, HAdV-D8 patients were rather old (median 69 years) and patients of these age group may have been exposed to HAdV-D8 many years ago but immunity may have waned. Therefore, the high age of the HAdV-D8 patients during the epidemic period can rather lead to the speculation that nosocomial transmission at routine visits to ophthalmologists’ offices (e.g. for glaucoma screening, presbyopia) may have contributed to the epidemic. However, this hypothesis is only supported by data on the first healthcare associated outbreak of the epidemic in Northrhine-Westphalia in late 2011^13^.

In conclusion, a prolonged multi-state HAdV-D8 epidemic was demonstrated by phylogenetic analysis of complete genomic sequences. Emergence of a novel monophyletic HAdV-D8 strain was observed in Germany starting 2010. However, co-circulation of other HAdV types as well as better reporting due to increased public awareness most likely have contributed to the huge increase of reported cases.

## Methods

### Description of cases reported to Robert Koch Institute, 2001–2015

All sporadic and outbreak-related cases reported to RKI in the period of 1 January 2001 until 31 December 2015 according to the Protection against Infection Act were included (datasource: “SurvNet@RKI”, accessed 29.01.2016). All cases fulfilled one of the RKI reference definitions:

- Cases with the clinical picture defined as redness of the conjunctiva AND with laboratory confirmation by direct detection of pathogens in conjunctival swabs using antigen detection (e.g. ELISA, IFT), pathogen isolation (cultural) or nucleic acid detection by PCR.
- Cases with the clinical picture defined as redness of the conjunctiva AND with an epidemiological link to a laboratory-proven infection in humans by human-to-human transmission or common source of exposure (e.g. ophthalmological examination devices). The incubation period of 5–12 days, occasionally longer, has to be taken into account.

Thus, in an outbreak at least one case has to be laboratory confirmed.

We also describe subgroups of cases with typing results reported to RKI in the period from November 2011 until December 2013 (epidemic period) as well as cases outside the epidemic period (2001 to October 2011, 2014 and 2015).

All statistical analyses were performed using STATA 14.0 for Windows (StataCorp LP, 4905 Lakeway Drive, College Station, TX 77845, USA).

### Description of cases with samples examined in the national reference laboratory for HAdV (KLA), 2001–2015

We describe all HAdV positive eye swab specimens submitted for laboratory analysis (HAdV detection and molecular typing) to the national reference laboratory for HAdV (“Konsilarlabor”, KLA) in the epidemic period as well as outside the epidemic period.

### Laboratory Methods used in the national reference laboratory for HAdV (KLA)

#### HAdV DNA quantification and molecular typing

HAdV DNA detection and molecular typing was performed on all eye swabs sent to the KLA. Viral DNA was extracted from eye swabs with the Qiagen blood kit (Qiagen, Hilden, Germany). A generic, real-time PCR for HAdV was performed on the DNA extracted directly from eye swabs as described previously^21^,^22^. Molecular typing by sequencing of the loop 2 of the major neutralization determinant ε was performed as described previously^23^. Additional virus isolation was performed using A549 (ATCC, CCL-185) cell cultures.

#### Next Generation Sequencing (NGS)

Complete genomic sequencing was performed from both cell culture isolates and DNA extracted directly from eye swabs as described previously^1^,^18^. Depending on the concentration of the input sample, library preparation was performed using either the Nextera or NexteraXT DNA Sample Prep Kits (Illumina, San Diego, CA) according to manufacturer’s protocol. Briefly, a total input of 50ng (Nextera kit) or 1ng (NexteraXT) from each DNA sample was fragmented and tagged with adaptors in the presence of transposomes, purified and enriched using a 5-cycle PCR (or 12-cycles in case of NexteraXT). Size selection and quality control was performed as described previously^18^. Samples were sequenced with an Illumina MiSeq generating paired-end 300 bp reads. The minimum achieved average coverage was 61 ± 13 fold and the highest average coverage achieved was 14206 ± 3495 fold. The data sets were assembled *de novo* and also using a genomic HAdV-D8 GenBank sequence (#AB746853.1) as a backbone. All data analysis was performed using the CLC Genomics Workbench version 7 (Aarhus, Denmark).

#### Phylogenetic Analysis

Whole genomic nucleotide sequences were aligned using the MAFFT online server^24^. The multiple alignment was visualized and edited using the BioEdit package (version 7.2). Phylogenetic analysis was performed using the MEGA software package (version 6). The phylogenetic tree from whole genomic nucleotide sequences was constructed using the Neighbor-joining algorithm. Kimura 2-parameter was used as a substitution model with 1000 bootstrap replicates.

Restriction enzyme analysis (REA) was performed *in silico* for the enzymes BamHI, HindIII, PstI, SalI, SmaI, and SacI cutting sites using the CLC Genomics Workbench version 7 (Aarhus, Denmark).

#### Ethical statement

All methods were carried out in accordance with relevant guidelines and regulations. For scientific use of routine anonymized data, ethical approval is not required in Germany (confirmed by the Ethikkommission of the Medizinische Hochschule Hannover (2586–2015)). Informed consent of patients is not required for this type of study.

## Additional Information

**Accession Codes:** The HAdV-D8 genomic sequences produced in this study have been deposited under the following accession numbers: KP016719- KP016743 and KT862545-KT862549.

**How to cite this article**: Hage, E. *et al*. Molecular phylogeny of a novel human adenovirus type 8 strain causing a prolonged, multi-state keratoconjunctivitis epidemic in Germany. *Sci. Rep.* **7**, 40680; doi: 10.1038/srep40680 (2017).

**Publisher's note:** Springer Nature remains neutral with regard to jurisdictional claims in published maps and institutional affiliations.

## Figures and Tables

**Table 1:** Adenovirus keratoconjunctivitis outbreaks and cases reported to RKI, 2001–2015.

Year	Outbreaks	Cases in outbreaks	Sporadic cases	Total cases
2001	3	7 (54)	71	132
2002	3	5 (2)	74	81
2003	5	14 (297)	86	397
2004	42	69 (464)	125	658
2005	6	38 (11)	89	138
2006	25	69 (165)	344	578
2007	16	25 (111)	239	375
2008	10	23 (24)	133	180
2009	7	10 (13)	146	169
2010	27	46 (92)	351	489
2011	25	60 (230)	383	673
2012	92	302 (734)	1110	2146
2013	77	197 (296)	1493	1986
2014	36	129 (136)	902	1167
2015	20	48 (52)	463	563
Total	394	1042 (2681)	6009	9732

Outbreaks are counted in the year of the first case in the outbreak. Cases counted as epidemiologically linked do not have laboratory confirmation and numbers are given in brackets.

**Table 2:** Outbreaks, outbreak cases and sporadic cases of Adenovirus-Keratoconjunctivitis reported to RKI with laboratory confirmation (lab) by typing (HAdV-D8, HAdV-D64, other HAdV types), nucleic acid detection (PCR), or other laboratory methods (e.g. antigen detection, virus isolation).

	HAdV-D8	HAdV-D64	HAdV other types	Nucleic acid detection (without typing)	Other laboratory methods (without typing)	Total
2001–Oct. 2011						
outbreaks [n]	3	12	0	90	55	160
outbreak cases [lab (epi)]	32 (26)	31 (53)	0 (0)	200 (895)	86 (280)	349 (1254)
sporadic cases [lab]	7	102	23	1323	504	1959
Epidemic period*						
outbreaks [n]	14	1	1	157	5	178
outbreak cases [lab (epi)]	40 (318)	1 (37)	2 (3)	465 (743)	8 (138)	516 (1239)
sporadic cases [lab]	39	3	8	2406	229	2685
2014, 2015						
outbreaks [n]	0	0	0	45	11	56
outbreak cases [lab (epi)]	0 (0)	0 (0)	0 (0)	135 (133)	42 (55)	177 (188)
sporadic cases [lab]	1	2	9	1183	170	1365
Total 2001–2015						
outbreaks [n]	17	13	1	292	71	394
outbreak cases [lab (epi)]	72 (344)	32 (90)	2 (3)	800 (1771)	136 (473)	1042 (2681)
sporadic cases [lab]	47	107	40	4912	903	6009

For outbreak cases the respective epidemiologically linked cases are shown in brackets (epi).

^*^The epidemic period is defined from 1 November 2011 until 31 December 2013.

**Table 3:** Comparison of HAdV-D8-outbreak cases and other outbreak cases reported to RKI from January 2001 until December 2015 by demographic information and exposure.

	HAdV-D8 outbreak cases n = 416	Other outbreak cases n = 3307	Odds Ratio (OR)	p-value*
Median age (interquartile range)	65 (47.5–75)	50 (22–70)		0.000
Female (%)	237 (57.1)	1677 (50.8)	1.3 (1.05–1.59)	0.015
Health care associated** (%)	233 (98.7)	340 (28.6)	193 (61.5–608)	0.000
Cases observed during epidemic period (%)	358 (86.1)	1397 (42.2)	8.4 (6.3–11.2)	0.000

^*^Wilcoxon rank sum test.

^**^Infection presumably acquired in hospital or medical treatment center (note: only 236 and 1187 cases with information on the setting, respectively).
